# Phenotypical characteristics of nontuberculous mycobacterial infection in patients with bronchiectasis

**DOI:** 10.1186/s12931-024-02904-0

**Published:** 2024-07-15

**Authors:** Assaf Frajman, Shimon Izhakian, Ori Mekiten, Ori Hadar, Ariel Lichtenstadt, Chen Hajaj, Shon Shchori, Moshe Heching, Dror Rosengarten, Mordechai R. Kramer

**Affiliations:** 1 Pulmonary Institute, Rabin Medical Center – Beilinson Hospital, 39 Jabotinksy St, Petach Tikva, 4941492 Israel; 2 Faculty of Medicine, Tel Aviv University, Tel Aviv, 6997801 Israel; 3 The Adelson School of Medicine, Ariel University, Ariel, 4076414 Israel; 4 Industrial Engineering and Management, Ariel University, Ariel, 40700 Israel

**Keywords:** Nontuberculous mycobacteria, Infection, Bronchiectasis, Pulmonary infection, Phenotype

## Abstract

**Background:**

The global mortality and morbidity rates of bronchiectasis patients due to nontuberculous mycobacteria (NTM) pulmonary infection are on a concerning upward trend. The aims of this study to identify the phenotype of NTM-positive individuals with bronchiectasis.

**Methods:**

A retrospective single-center observational study was conducted in adult patients with bronchiectasis who underwent bronchoscopy in 2007-2020. Clinical, laboratory, pulmonary function, and radiological data were compared between patients with a positive or negative NTM culture.

**Results:**

Compared to the NTM-negative group (*n*=677), the NTM-positive group (*n*=94) was characterized (*P* ≤0.05 for all) by older age, greater proportion of females, and higher rates of gastroesophageal reflux disease and muco-active medication use; lower body mass index, serum albumin level, and lymphocyte and eosinophil counts; lower values of forced expiratory volume in one second, forced vital capacity, and their ratio, and lower diffusing lung capacity for carbon monoxide; higher rates of bronchiectasis in both lungs and upper lobes and higher number of involved lobes; and more exacerbations in the year prior bronchoscopy. On multivariate analysis, older age (OR 1.05, 95% CI 1.02-1.07, *P*=0.001), lower body mass index (OR 1.16, 95% CI 1.16-1.07, *P* <0.001), and increased number of involved lobes (OR 1.26, 95% CI 1.01-1.44, *P*=0.04) were associated with NTM infection.

**Conclusions:**

Patients with bronchiectasis and NTM pulmonary infection are more likely to be older and female with more severe clinical, laboratory, pulmonary function, and radiological parameters than those without NTM infection. This phenotype can be used for screening patients with suspected NTM disease.

## Background

Bronchiectasis is a chronic progressive lung disorder characterized by irreversible bronchial dilation, leading to repeated pulmonary infections and chronic inflammation. The incidence of bronchiectasis is increasing worldwide [1–3], accompanied by a growing socioeconomic burden and increased morbidity and mortality [3–5].

The incidence of lung infection caused by nontuberculous mycobacteria (NTM), ubiquitous in the environment, is rising globally as well [6, 7]. Patients with bronchiectasis are particularly susceptible [8, 9] because their impaired muco-ciliary clearing mechanism inhibits an important natural protective barrier against NTM [3]. The diagnosis of NTM pulmonary infection based on the clinical presentation alone can be challenging in patients with bronchiectasis owing to the similar pulmonary symptoms to patients without NTM [10, 11]. Radiological findings may be instructive, as the presence of NTM pulmonary infection is often associated with more extensive lung involvement [10–14]. In their meta-analysis, Zhu et al. [10] concluded that the significant heterogeneity among studies of clinical markers of NTM isolation in bronchiectasis may limit the diagnostic utility of these markers, particularly as some heterogeneity may be traced to geographic variations. Evidence-based associations between clinical markers and NTM infections would equip clinicians with a tool for early identification of patients requiring NTM cultures as well as close follow-up. The aim of the present study was to describe the phenotype of NTM-positive patients with bronchiectasis in Israel.

## Patients and methods

### Study population and design

A retrospective study was performed in adult patients (age ≥18 years) with bronchiectasis who underwent bronchoscopy including NTM cultures between January 2007 and August 2020 at a single tertiary university medical center in Israel. Bronchoscopy was indicated for patients with bronchiectasis exacerbation who failed to respond to empiric therapy and showed no evidence of a pathogen, either due to no sputum production or failure of sputum cultures to grow a pathogen. Exclusion criteria were cystic fibrosis, no available mycobacterial cultures, and active tuberculosis at the time of bronchoscopy. The study was conducted in accordance with the Declaration of Helsinki and was approved by institutional review board of the Rabin Medical Center.

### Data collection

Data for the study were obtained at the time of bronchoalveolar lavage (BAL) from the electronic medical record database system which integrates medical information from all hospitals in Israel. The following variables were recorded: age, sex, body mass index, comorbid conditions, number of exacerbations in the past, serum albumin level, complete blood count, pulmonary function tests, radiological location of the bronchiectasis, and results of bacterial and fungal cultures. The patients were divided into two groups for analysis: positive NTM cultures and no positive NTM cultures.

### Bronchoscopy procedure

All bronchoscopy procedures were performed at the hospital's pulmonary institute during exacerbation of bronchiectasis. To prevent contamination by upper airway flora, the trap used to collect the specimen was connected to the suction channel of the bronchoscope only after the bronchoscope traversed the vocal cords. The bronchoscope was wedged into a subsegmental bronchus (usually toward the place of infiltrate, when present), and 3 aliquots of sterile saline (50 mL each) were instilled and aspirated.

### Microbiologic samples

Bronchoalveolar samples were plated on blood, chocolate, and MacConkey agars. All specimens were analyzed for mycobacteria using Ziehl-Neelsen stain and cultured on Lowenstein medium and in Mycobacterium Growth Indicator Tubes (BACT MGIT 960, Becton Dickinson Baltimore, MD, USA). Negative bacterial cultures were discarded after 7 days, and MGIT and Lowenstein cultures were discarded after 6-8 weeks. The GenoType Mycobacterium DNA strip assay (Hain Lifescience GmbH, Negren, Germany) was used to detect and identify the species of mycobacteria obtained from positive liquid and solid mycobacterial cultures. Quantitative NTM cultures are not performed in our center.

### Definitions

The diagnosis of bronchiectasis was based on clinical (chronic cough and sputum production, and history of exacerbations) and radiological (presence of bronchiectasis on chest computed tomography scan) criteria [3, 15]. An exacerbation was defined as worsening of the following symptoms for at least 48 hours: cough, sputum volume and/or consistency, sputum purulence, breathlessness and/or exercise intolerance, fatigue and/or malaise, and hemoptysis, and/or determination by a clinician that antibiotic treatment was required [3, 16]. NTM pulmonary infection was diagnosed on the basis of a single positive culture obtained by bronchoscopy [17, 18].

### Statistical analysis

Descriptive data were expressed as mean and standard deviation or number and percentage of the presented cases. Chi-square test was used to compare categorical variables between groups, and Student’s t-test was used to compare continuous variables. *P* ≤0.05 was considered statistically significant. Multivariate analysis (logistic regression model) was performed to identify the variables most significantly associated with NTM pulmonary infection. Statistical analyses were performed using SAS software, version 9.4 (SAS Institute Inc.).

## Results

### Patient characteristics

**Fig. 1:**
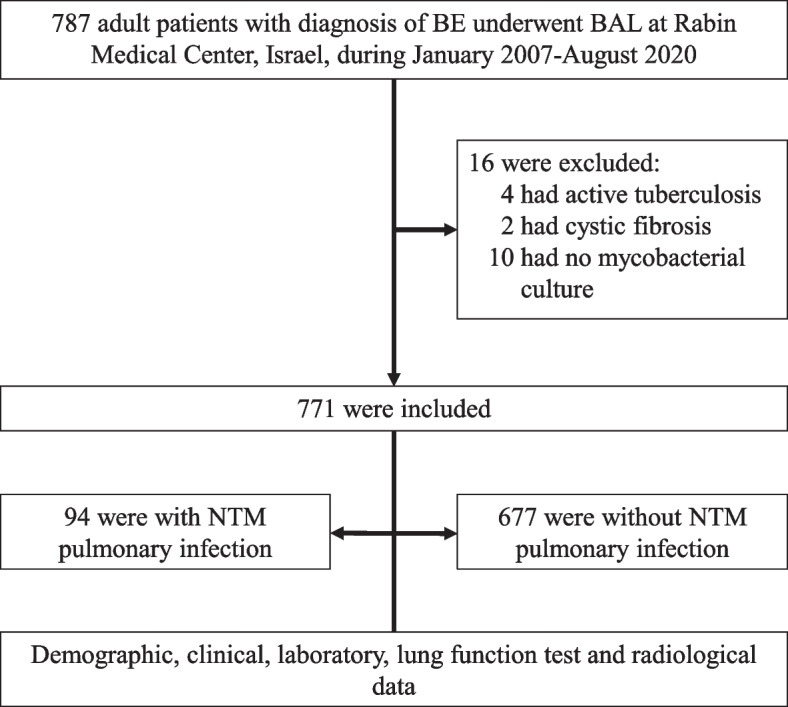
Flow chart presenting the study design. Abbreviations: BAL: bronchoalveolar lavage; NTM: nontuberculous mycobacteria

Of 787 adult patients with bronchiectasis who underwent bronchoscopy during the study period, 16 were excluded from the analysis: 2 with cystic fibrosis, 10 with no available mycobacterial cultures, and 4 with active tuberculosis at the time of bronchoscopy (Fig. 1). Table 1 presents the demographic and clinical characteristics of the remaining 771 patients. Mean age was 61.11±16.31 years; 61.4% were female. The cultures obtained by bronchoscopy were positive for NTM in 94 patients (12.2%) and negative in 677. Comparison of the groups revealed that the patients with pulmonary NTM infection were older (*P*=0.008) and more likely to be female (*p*=0.04), and were more often treated with muco-active agents (*P*=0.01). They had a lower mean body mass index (*P* <0.001) and a higher incidence of gastroesophageal reflux disease (*P*=0.05) and experienced more exacerbations in the year prior to the bronchoscopy (*P*=0.02).

**Table 1:** Demographic and clinical characteristics of patients with bronchiectasis, whole cohort and stratified by presence/absence of NTM infection

**Variable**	**Entire sample (*n*=771)**	**Non-NTM group (*n*=677)**	**NTM group (*n*=94)**	*p* value*
Age (years)	61.1±16.3	60.5±16.5	65.3±14.3	**0.008**
Female sex	473 (61.4%)	406 (60.0%)	67 (71.3%)	**0.04**
Body mass index (kg/m^2^)	24.6±5.0	24.9±5.1	22.5±3.7	**<0.001**
Prior or current somking	232 (31.4%)	210 (31.0%)	32 (34.0%)	0.81
Comorbid conditions				
Hypertension	271 (35.2%)	240 (35.5%)	31 (33.0%)	0.56
GERD	248 (32.2%)	208 (30.7%)	40 (42.6%)	**0.05**
Allergy	152 (19.7%)	132 (19.5%)	20 (21.3%)	0.68
COPD	150 (19.5%)	126 (18.6%)	24 (25.5%)	0.13
Asthma	136 (17.6%)	119 (17.6%)	17 (18.1%)	0.89
Diabetes mellitus	134 (17.4%)	121 (17.9%)	13 (13.8%)	0.39
Chronic sinusitis or otitis	90 (11.7%)	82 (12.1%)	8 (8.5%)	0.39
Ischemic heart disease	84 (10.9%)	72 (10.6%)	12 (12.8%)	0.60
Cerebrovascular disease	52 (6.7%)	43 (6.4%)	9 (9.6%)	0.27
Rheumatic disease	52 (6.7%)	47 (6.9%)	5 (%.3%)	0.67
Malignant disease	33 (4.3%)	29 (4.3%)	4 (4.3%)	1.0
Interstitial lung disease	24 (3.1%)	20 (3.0%)	4 (4.3%)	0.52
Immunodeficiency disease	24 (3.1%)	22 (3.3%)	2 (2.1%)	0.76
Inflammatory bowel disease	22 (2.9%)	19 (2.8%)	3 (3.2%)	0.74
History of tuberculosis	17 (2.2%)	14 (2.1%)	3 (3.2%)	0.46
Primary ciliary dyskinesia	11 (1.4%)	10 (1.5%)	1 (1.1%)	1.0
Allergic bronchopulmonary aspergillosis	7 (0.9%)	5 (0.7%)	2 (2.1%)	0.21
Treatment				
Inhaled corticosteroids	207 (26.9%)	176 (26.0%)	31 (33.0%)	0.26
Long-acting beta-agonists	207 (26.9%)	174 (25.7%)	33 (35.1%)	0.10
Long-acting muscarinit antagonists	53 (6.9%)	42 (6.2%)	11 (11.7%)	0.08
Antibiotics (any kind in the past year)	57 (7.4%)	47 (6.9%)	10 (10.6%)	0.29
Systemic corticosteroids (past year)	51 (6.6%)	45 (6.7%)	6 (6.4%)	1.0
Mucoactive agents	57 (7.4%)	43 (6.4%)	14 (14.9%)	**0.01**
Number exacerbations/patient in the year preceding BAL	0.9±1.0	0.9±0.0	1.2±1.0	**0.02**

*NTM* Nontuberculous mycobacteria, *GERD* Gastroesophageal reflux disorder, *COPD* Chronic obstructive pulmonary disease, *BAL* Broncheolar lavage

Data are presented as mean±standard deviation or number (percentage) of presented cases

^*^Difference between the NTM and non-NTM groups

Bold entries in the table idicate a *P*-value of ≤0.05

The laboratory and radiological data of the study patients are presented in Table 2. The NTM-positive group was characterized by a significantly lower mean serum albumin level (*P*=0.02), lymphocyte count (*P*=0.02) and eosinophil count (*P*=0.05) than the NTM-negative group. They exhibited lower mean absolute values of forced expiratory volume in one second (FEV1, *P*=0.01) and forced vital capacity (FVC, *P*=0.04), as well as a lower mean FEV1/FVC ratio (*P*=0.04) and a lower percentage of predictive value of carbon monoxide diffusion capacity (DLCO, *P*=0.02). The NTM-positive group had a higher proportion of patients in whom the bronchiectasis was located in the upper lobes (*P* <0.001), lingula (*P*=0.004) and both lungs (*P*=0.002), and a higher mean number of involved lobes (*P* <0.001).

**Table 2:** Laboratory, lung function test and radiological data of patients with bronchiectasis, whole cohort and stratified by presence/absence of NTM infection

**Variable**	**Entire sample (*n*=771)**	**Non-NTM group (*n*=677)**	**NTM group (*n*=94)**	*p*-value*
Laboratory data				
Serum creatinine (normal 0.5-0.9 mg/dl)	0.80±0.3	0.80±0.3	0.79±0.4	0.12
Serum albumin on admission (normal 34-48 g/l)	41.0±4.0	41.1±4.0	40.2±3.4	**0.02**
Blood hemogloblin (normal 13.0-16.2 g/dl)	12.9±1.5	13.0±1.5	12.8±1.4	0.26
Red cell distribution width (normal 12.0-14.7%)	14.1±15	14.1±1.4	14.2±1.7	0.93
Platelet count (normal 140-450x10^9^/l)	259±88	258±87	267±95	0.43
Mean platelet volume (normal 7.3-11.5 gL)	9.5 ±.4	9.5±1.4	9.6±1.5	0.36
White blood cell count (normal 4.0-11.0x10^9^/l)	7.7±5.2	7.8±5.5	7.0±2.1	0.15
Neutrophil count (normal 2.0-7.7x10^9^/l)	4.8±2.3	4.8±2.3	4.6±1.8	0.96
Lymphocyte count (normal 1.0-4.0x10^9^/l)	2.2±4.5	2.2±4.8	1.6±0.6	**0.02**
Monocyte count (normal 0.2-0.8x10^9^/l)	0.48±0.2	0.47±0.2	0.52±0.2	0.06
Eosinophil count (normal 0-0.5x10^9^/l)	0.21±0.2	0.22±0.2	0.16±0.1	**0.05**
Lung function test				
FEV1 (liters)	1.8±0.7	1.9±0.8	1.6±0.7	**0.01**
FEV1 (% of predicted value)	75.1±24.1	75.7±23.8	71.5±26.0	0.15
FVC (liters)	2.5± 0.9	2.5±0.9	2.3±0.8	**0.04**
FVC (% of predicted value)	82.6±22.3	82.7±22.0	82.2±24.3	0.77
FEV1/FVC ratio	0.73±0.1	0.7±30.1	0.70±0.1	**0.04**
TLC (% of predicted value)	97.1±17.9	96.8±17.4	98.8 ±20.7	0.49
RV (% of predicted value)	135.8±42.2	134±41.6	142±46.3	0.39
DLCO (% of predicted value)	79.3±21.3	80.4±20.8	72.4±23.6	**0.02**
Radiological location of bronchiectasis				
Left upper lobe	101 (13.1%)	72 (10.6%)	29 (30.9%)	**<0.001**
Left lower lobe	281 (36.5%)	242 (35.8%)	39 (41.5%)	0.91
Lingula	220 (28.5%)	176 (26.0%)	44 (46.8%)	**0.004**
Right upper lobe	157 (20.4%)	111 (15.4%)	46 (48.9%)	**<0.001**
Right middle lobe	338 (43.8%)	283 (41.8%)	55 (58.5%)	0.14
Right lower lobe	262 (34.0%)	220 (32.5%)	42 (44.7%)	0.30
Any upper lobe	194 (25.2%)	144 (21.3%)	50 (53.2%)	**<0.001**
Both lungs	338 (43.8%)	275 (40.6)	63 (67.0%)	**0.002**
Number of involved lobes	2.1±1.3	2.0±1.2	2.8±1.7	**<0.001**

Data are presented as mean±standard deviation or number (percentage) of presented cases

^*^Difference between the NTM and non-NTM groups

Bold entries in the table idicate a *P* -value of ≤0.05

*NTM* nontuberculous mycobacteria; *FEV1* forced expiratory volume in one second; *FVC* forced vital capacity; *TLC* total lung capacity; *RV* residual volume; *DLCO* diffusing lung capacity for carbon monoxide

Data on the BAL cultures that were positive for bacteria and fungi are presented in Table 3. *Pseudomonas* sp. and *Haemophilus* sp. were the most common microorganisms grown. The only statistically significant difference between the NTM-positive and NTM-negative groups was the percentage of *Staphylococcus aureus* isolates (17.0% vs. 8.6%, respectively, *P*=0.01).

**Table 3:** Data of positive BAL cultures of patients with bronchiectasis, whole cohort and stratified by presence/absence of NTM infection

**Variable**	**Entire sample (*n*=771)**	**Non-NTM group (*n*=677)**	**NTM group (*n*=94)**	*p* value*
Any positive bacterial culture in the previous year	430 (55.8%)	377 (55.7%)	53 (56.4%)	0.91
*Haemophilus* sp.	140 (18.6%)	129 (19.1%)	11 (11.7%	0.09
*Pseudomonas* sp.	105 (13.6%)	88 (13.0%)	17 (18.1%)	0.20
*Staphylococcus aureus*	74 (9.6%)	58 (8.6%)	16 (17.0%)	**0.01**
*Streptococcus pneumoniae*	45 (5.8%)	40 (5.9%)	5 (5.3%)	1.0
Any positive fungal culture in the previous year	65 (8.4%)	57 (8.4%)	8 (8.5%)	1.00
*Aspergillus* sp.	41 (5.3%)	37 (5.5%)	4 (4.3%)	0.81
*Aspergillus* sp.	23 (3.0%)	19 (2.8%)	4 (4.3%)	0.51

*BAL* Bronchoalveolar lavage, *NTM* Nontuberculous mycobacteria

Data are presented as number (percentage) of presented cases

^*^Difference between the NTM and non-NTM gropus

Bold entries in the table indicate a *P*-value of ≤0.05

### Spectrum of NTM pulmonary infection

The following NTM sp. were identified in the 94 affected patients (in some patients, more than one type of NTM was isolated): *Mycobacterium avium complex* (MAC, *n*=44), *M. simiae* (*n*=29), *M. kansasii* (*n*=11), *M. abscessus* (*n*=6), *M. fortuitum* (*n*=3), *M. szulgai* (*n*=1), *M. chelonae* (*n*=1), *M. gordonae* (*n*=1) and *M. shimoidei* (*n*=1).

### NTM pulmonary disease

In a study of 32 patients with NTM pulmonary disease, the leading presenting complaints were dyspnea (*n*=28), chronic cough (*n*=26), chronic sputum production (*n*=20), weight loss (*n*=5), fever (*n*=4), and chest pain (*n*=5). All patients underwent high-resolution computed tomography (HRCT), with the predominant findings being pulmonary cavitary lesions (*n*=20), tree-in-bud pattern (*n*=26), nodules (*n*=28), and mucus plugging (*n*=17).

### Multivariate analysis

On multivariate analysis, the following variables were most significantly associated with NTM pulmonary infection in the entire bronchiectasis cohort: older age [odds ratio (OR) 1.05, 95% confidence interval (CI) 1.02-1.07, *P*=0.001), lower body mass index (OR 1.16, 95% CI 1.16-1.07, *P* <0.001) and higher number of involved lobes (OR 1.26, 95% CI 1.01-1.44, *P*=0.04).

## Discussion

This study describes the phenotype of patients with NTM-positive bronchiectasis in Israel. We found that among the whole cohort with bronchiectasis, the patients with positive NTM cultures were older than the patients with negative NTM cultures, tended to be female, and had indicia of advanced disease including lower body mass index, more extensive bronchiectasis, and a greater propensity for exacerbations.

Our findings are consistent with prior studies. Patients with NTM pulmonary infection are generally characterized by older age [11–13, 19] which may entail an age-dependent decrease in respiratory muscle strength and mucocilliary clearance and an association with other factors such as impaired cell-mediated immunity, cytokine production, and phagocytosis [20]. The pathophysiologic mechanisms underlying the association of NTM infection and female gender, also reported by others [11, 12, 21], are not fully understood. It is noteworthy that there is a similar gender bias of bronchiectasis in general. Possible explanations include age-related deficiency of estradiol in women [20] and a higher prevalence of autoimmune disorders requiring immunosuppressive treatment among females than males with bronchiectasis that may contribute to susceptibility to NTM.

Similarly, the association of lower BMI with NTM pulmonary infection has been previously reported [11, 12, 19, 21]. While lower BMI may simply be a result of the NTM infection itself, it has been suggested that decreased fat mass with corresponding regulatory dysfunction of adipokines that participate in the immune response, such as leptin and adiponectin, may increase susceptibility to NTM infection [21, 22]. Moreover, we observed a relatively greater reduction in serum albumin among patients in the NTM-positive group, attributable to reduced protein synthesis, accelerated catabolism, and altered body distribution of albumin resulting from the combined effects of NTM infection, inflammation, and impaired nutritional status [22]. Both lower BMI and hypoalbuminemia are associated with disease progression [22] and mortality [23].

Among the various comorbidities, we identified an association of NTM infection with GERD, noted in an earlier study as well [12]. While GERD may contribute to the progression of bronchiectasis due to repeated insult of refluxed gastric contents to the bronchial tree [24], NTM pulmonary disease may predispose patients to GERD because of the frequent coughing and impaired mucus clearance [24].

One of the novel findings of our study was the more frequent use of muco-active medications by patients who had NTM pulmonary infection compared to those who did not. By contrast, the single pertinent study in the literature reported more common muco-active therapy in patients free of NTM pulmonary infection [12]. The reason for this discrepancy is unclear and may be related to differences in study design or in the prevalence of the relevant comorbidities. It is difficult to conclude on the basis of our data whether NTM-infected patients with bronchiectasis are more likely to need muco-active treatment due to excessive mucus production or whether the increased muco-ciliary secretion associated with bronchiectasis makes these patients more susceptible to NTM infection.

Of note, patients in the NTM group had more exacerbations in the 12 months prior to bronchoscopy, in agreement with the findings of Yin et al. [11]. However, Aksamit et al. [12] noted a lower incidence of exacerbations leading up to diagnosis in patients with NTM infection. This issue warrants further investigation.

The main novel finding of the present study was the association of NTM infection in patients with bronchiectasis with lower values of several routine laboratory parameters, such as serum albumin and blood counts of lymphocytes and eosinophils. Importantly, the presence of hypoalbuminemia in patients with NTM lung disease has been reported to predict disease progression [22] and mortality [23]. The lower levels of serum albumin in our NTM-positive patients may be explained mainly by reduced synthesis, accelerated catabolism, and altered body distribution of albumin resulting from the combined effects of a more severe infection, inflammation, and poor nutritional status [22].

Regarding the lower counts of lymphocytes and eosinophils, to our knowledge, no studies of patients with bronchiectasis to date compared these parameters between those who were positive or negative for NTM infection. While lymphopenia likely reflects a decreased host immune and nutritional status, the lymphopenia in MAC lung disease has been associated with recurrence following treatment [25] and a worse prognosis [26]. The pathogenesis of the lower eosinophil counts in the NTM-positive group is unclear. We suggest that it may indicate a more severe clinical status compared to patients without NTM infection. Notably, a previous study of a patient population with bronchiectasis found that a higher eosinophil count (>1.0x109/l) was associated with better nutritional status, lung function, and clinical outcomes [27].

An additional interesting observation in our investigation was the significantly higher isolation rate of *Staphylococcus aureus* from BAL cultures in the NTM-positive than the NTM-negative group (17.0% vs. 8.6%) relative to the other bacteria and fungi evaluated for which isolation rates were comparable in the two groups. Respiratory culture data are scarce in patients with bronchiectasis with and without NTM pulmonary infection [10]. Only a single study reported positive cultures rates for *Staphylococcus aureus*, and in contradiction to our results, they were significantly lower in the patients with NTM infection than in those without (10.0% and 15.0%, respectively).

Moreover, spirometry values (FEV1, FVC) and DLCO were decreased in the patients with NTM pulmonary infection. This finding is likely attributable to their more the extensive bronchiectasis and the attendant destruction of the lung parenchyma compared to the NTM-negative group. Similarly, others reported an association of NTM infection with a greater decline in spirometry overtime [28], more extensive radiological findings, and involvement of multiple lobes [10–14].

Finally, we found no statistically significant association between inhaled corticosteroids (ICS) and NTM infection. This finding contrasts with other research reports that show an association between high-dose ICS inhaler usage and acquiring NTM infection [29, 30]. Several explanations for this discrepancy may exist. First, our findings may be influenced by the criteria we used for diagnosing NTM pulmonary infection rather than NTM pulmonary disease. Second, the small sample size of the NTM group may have limited our ability to detect a significant difference. It is worth noting a trend towards a higher proportion of NTM culture-positive results in the ICS group (33% vs. 26%), which may become significant with a larger sample size. Third, a recent meta-analysis and systematic review found that the correlation between ICS and NTM infection was especially significant in the higher-dose ICS group. In our study, we did not perform a subanalysis based on ICS dose [31].

The strengths of our study are the large sample of cases and the diagnosis of NTM infection on the basis of BAL, performed in all patients. In addition, the evaluated data included wide spectrum of variables which made it possible to determine the NTM infection risk.

The limitations of the study include its retrospective, observational design, and single-center setting. We only included patients experiencing bronchiectasis exacerbation who were unresponsive to empiric therapy and lacked evidence of a pathogen, which represents a specific patient population and may restrict the generalizability of the results.

## Conclusion

NTM-infected patients with bronchiectasis in Israel are older, more likely to be female, and have more severe clinical, laboratory, pulmonary function, and radiological characteristics than patients with bronchiectasis without NTM infection. Based on multivariate analysis, the most clinically significant markers of NTM infection are older age, low body mass index, and radiological extent of bronchiectasis. This phenotype can be used by clinicians for screening patients with suspected NTM disease.

## Data Availability

No datasets were generated or analysed during the current study.
